# Controllable growth of three-dimensional CdS nanoparticles on TiO_2_ nanotubes to enhance photocatalytic activity†

**DOI:** 10.1039/c9ra10895e

**Published:** 2020-04-29

**Authors:** Guo-Min Liu, Wen-Yuan Jia, Qiu-Shi Jiang, Zhi-Qiang Cheng

**Affiliations:** Department of Orthopedics, The Second Hospital of Jilin University Changchun 130000 China; College of Resources and Environment, Jilin Agriculture University Changchun 130118 China czq5974@163.com

## Abstract

Exploiting photocatalysts with characteristics of low cost, high reactivity and good recyclability is a great significance for environmental remediation and energy conversion. Herein, hollow TiO_2_ nanotubes were fabricated by a novel and efficient method *via* electrospinning and an impregnation calcination method. With the hydrothermal method, the CdS nanoparticles were modified on the surface and in walls of the TiO_2_ nanotubes. By changing the reaction conditions, the morphology of CdS nanoparticles presents a controllable three-dimensional (3D) structure. The morphology of the samples was characterized by scanning electron microscopy (SEM) and transmission electron microscopy (TEM). The structure and components of samples were characterized by X-ray diffraction (XRD), energy dispersive X-ray analysis (EDX) and X-ray photoelectron spectroscopy (XPS). The light absorption efficiency was detected using UV-vis diffuse reflectance spectroscopy (DRS) and photoluminescence (PL). The photocatalytic properties were evaluated by degradation of methyl orange (MO) and photocatalytic hydrogen evolution under visible light irradiation. From the results, the TiO_2_/CdS nanotubes exhibit better photocatalytic activity than the pure TiO_2_. The synthetic mechanism of TiO_2_/CdS heterostructures and a possible photocatalytic mechanism based on the experimental results were proposed.

## Abstract 2

Exploiting photocatalysts with characteristics of low cost, high reactivity and good circularity is a great significance for environmental remediation and energy conversion.

## Graphical abstract

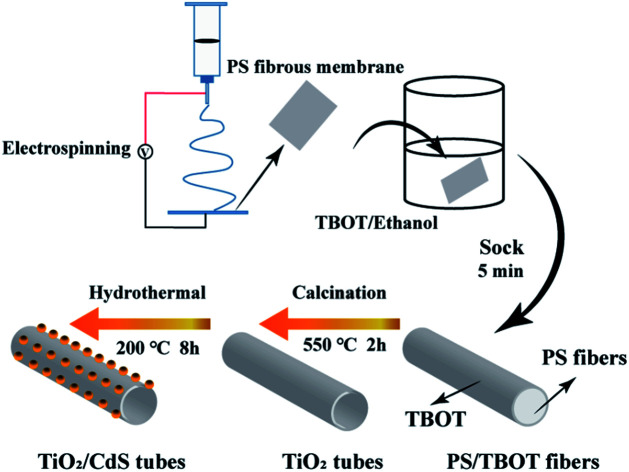

## 1 Introduction

With progress in the field of nanotechnology, it has become possible to prepare semiconducting materials of nanocrystal structures with special shapes and dimensions, such as nanofibers, nanorods, nanoflowers, nanospheres and nanotubes,^1–5^ which have garnered extensive attention on account of their unique electronic, optical, and photoactive properties.^6–9^ Meanwhile, they are widely utilized in the fields of gas sensors, optical devices, visible light detectors, and field emission emitters, especially for photocatalysis.^10–15^ Among copious inorganic semiconductor nanostructures, titanium oxide (TiO_2_) has been extensively studied to date, with extensive research on its high surface-to-volume ratio, low cost, environmental friendliness and unique physical and chemical properties.^16–19^ Recently, the application of TiO_2_ as photocatalysts for degradation of toxic organic pollutants have attracted wide public attention, because the TiO_2_ nanostructure and its composites can significantly improve the photocatalytic performance.^20–22^ The properties of nanomaterials mainly rely on their nanoscale and morphology, for nanoscale semiconductor to perform well as catalysts, they at least satisfy three characteristics: (1) high specific surface area, (2) have good stability and (3) the size and distribution should be uniform.^23–25^ Therefore, TiO_2_ nanotubes shows intrinsic advantages compared to traditional TiO_2_ in terms of size and specific surface area,^26^ design and fabricate TiO_2_ based nanotubes for heterogeneous photocatalysis have become a hot research topic. Thus far, many synthesis methods such as electrodeposition method, sol–gel method, template-assisted and hydrothermal treatment, have been developed for fabricating TiO_2_ nanotube.^18,19,27,28^ However, these traditional method often accompanied problems such as cumbersome operation, template hard removal, high production cost and poor structural stability, *etc.*^29^

Meanwhile, the high recombination rate of photogenerated (PG) electron/hole pairs is another limits factor of photocatalysis.^30,31^ Although reduce the size of nanoscale TiO_2_ open a convenient way for improving photocatalytic efficiency, but it cannot tackle the fundamental limits issue.^32,33^ By contrast, making TiO_2_ doped with other elements, modified with noble metal and combined with other semiconductors to form heterostructures, which provide a effective means to inhibit the recombination of PG electron/hole pairs.^34–38^ It is necessary to design new-type composite photocatalysts not only improve the separation of PG electron/hole pairs, but also extend the optical absorption of TiO_2_, thus to obtain a more efficient utilization of solar energy.^39,40^ Cadmium sulfide (CdS) is an excellent n-type semiconductor with an ideal band-gap energy of 2.4 eV, which could absorb the visible light radiation up to 520 nm,^41,42^ has been widely used as a visible light response sensitizer.^34,43^ By comparing the energy levels of TiO_2_ with CdS, which could find their analogical band structures are quite suitable to construct heterostructures and bring an effective separation of PG electron/hole pairs.^44,45^ Therefore, that is expected to combination the TiO_2_ with CdS form a heterostructure structure for overcoming the demerits that exist in TiO_2_ and CdS simultaneously.^46^

Herein, we described the fabrication of CdS decorated TiO_2_ nanotubes *via* electrospinning combined with controlled calcination and hydrothermal process. By changing the synthesis condition, the density and size of CdS on the surface (inner wall) of the TiO_2_ nanotubes can be controlled. The morphology and dimension of the CdS/TiO_2_ nanotubes were observed by the SEM and TEM. The crystal phases, optical absorption properties, elemental and chemical state were explored by XRD, DRS and XPS. Due to the formation of CdS/TiO_2_ heterojunction structure, the hybrid nanoparticles could reduce the recombination of PG electron–hole pairs, and the hollow nanotubes structure provides a larger specific surface area and more active sites, that the as-prepared CdS/TiO_2_ nanotubes significantly improved photocatalytic activity. Moreover, that may provide a new method for preparation of semiconductor heterostructure nanotubes.

## 2 Experimental

### 2.1 Materials

Polystyrene (PS, *M*_w_ ≈ 350 000 g mol^−1^), tetrabutyltitanate (TBOT, 97%), cadmium nitrate [Cd(NO_3_)_2_·4H_2_O, 99%], thiourea (CH_4_N_2_S, 99%), absolute ethanol (C_2_H_6_O, 99%), *N*,*N*-dimethylformamide (DMF, 99%), and methyl orange (MO, 96%) were purchased from Aladdin; all of chemicals used in the experiments were analytical grade and used without further purification.

### 2.2 Methods

The precursor solution was prepared by adding 1.4 g PS to 5.0 mL DMF with magnetic stirring at 50 °C. After PS dissolution in the DMF completely, the prepared solution was loaded into a syringe, which was connected to a 15 kV high voltage. The flow rate of the solution was controlled at 0.5 mL h^−1^, and the distance from needle to the rotate acceptor was 20 cm. The whole process was kept with humidity below 20%. Then, the PS membrane were soaked in the BOT/ethanol (volume ratio 1 : 10) precursor solution for 5 min. Then, the composite TBOT/PS precursor fibers were natural air dried. Finally, the precursor fibers were calcined to 550 °C with a rate of 2 °C min^−1^ and keep 550 °C for 2 hours, to burn the PS templates completely. After annealing, the hollow TiO_2_ nanotubes were obtained.

**Fig. 1:**
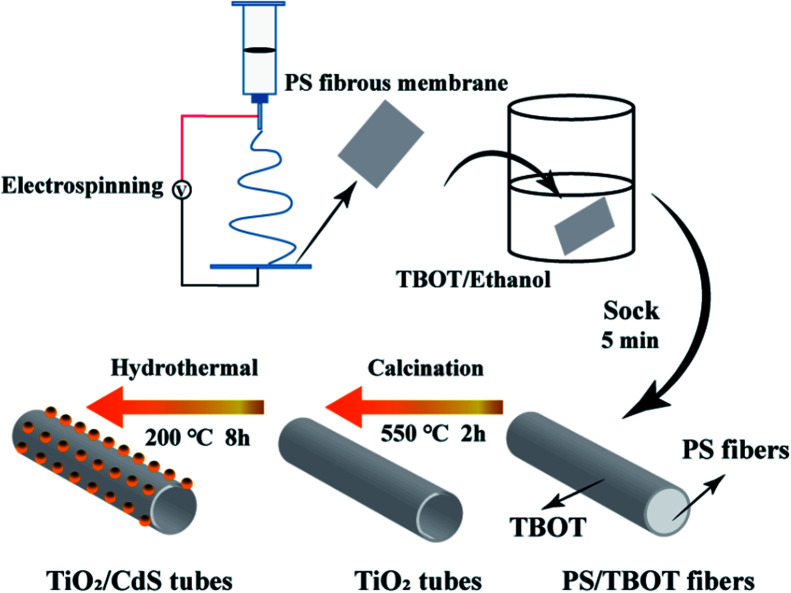
An illustration of the formation mechanism of the TiO_2_/CdS nanotubes.

Hydrothermal method was used for CdS nanoparticles modified. The as prepared TiO_2_ nanotubes were transferred into a Teflon-lined autoclave, which containing 30 mL solution of Cd(NO_3_)_2_ and thiourea, the concentration are equal and both volumes are 15 mL. Then, the Teflon-lined autoclave was heated to 200 °C and keep 8 hours. Finally, the CdS/TiO_2_ nanotubes were collected and washed in DI water. For compare the effect of reactant concentration on morphology, change the concentration of Cd(NO_3_)_2_ solution to 5, 10, 20, 30 mmol L^−1^ and the corresponding CdS/TiO_2_ nanotubes was named as TC1, TC2, TC3 and TC4, respectively (Fig. 1).

### 2.3 Measurement of photocatalytic activity

Methyl orange (MO) was used as the target substrates for evaluating the photocatalytic activity. The photocatalytic reaction was carried out in a reactor with a circulating water system to maintain a constant temperature. The xenon lamp (Philips, 100 W) equipped with a 420 nm cut-off filter instead and the irradiation time were ranged from 5 to 200 min. Under the same conditions, all samples were placed in the quartz tube, which away from the irradiation source 5 cm. The degradation ratio was 40 mL MO solution (10 mg L^−1^) with 40 mg photocatalyst. Then, the suspension was stirred in the dark for 1 hour to establish adsorption and desorption equilibrium. During the photoreaction process, approximately 3 mL of the mixture was sampled in a constant time, and use centrifugation to separate the nanoparticles. The, using the spectrophotometer to determination the solution concentration. H_2_ evolution reactions were proceed in a closed gas-circulation system. 50 mg samples were dispersed in 200 mL aqueous solution of Na_2_S (1.0 M) and Na_2_SO_3_ (1.0 M). After removing the air of reaction system, the solution were irradiated by a 100 W xenon lamp equipped with a 420 nm cut-off filter, and the amount of H_2_ were measured by gas chromatography (GC-9700, N_2_ carrier).

## 3 Results and discussion

### 3.1 XRD patterns

**Fig. 2:**
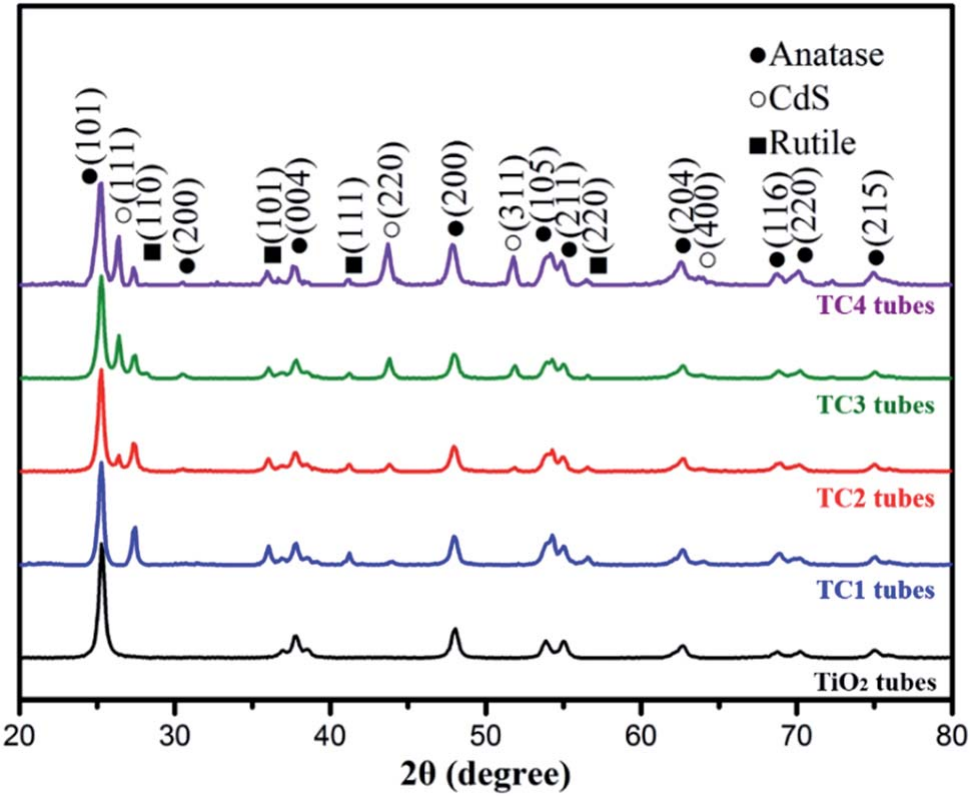
The XRD patterns of the as-prepared photocatalysts.

Fig. 2 present the XRD pattern of the as-prepared photocatalysts. The sharp and strong diffraction peaks showed the nanotubes were well crystallized. The main diffraction peaks of TiO_2_ were located at 25.45°, 37.95°, 48.20°, 55.16° and 62.81° can be indexed to the (101), (004), (200), (211) and (204) directions, respectively, which indicates that the prepared titanium oxide belongs to the anatase structure (JCPDS 21-1272), and with a high purity. Compared with pure TiO_2,_ the CdS/TiO_2_ nanotubes can observe the peak at 26.52°, 43.72° confirms the formation of CdS peaks (JCPDS 65-2280), and the peaks were enhanced with increased Cd^2+^ concentration. Meanwhile, there is also a certain amount of rutile phase at 27.2° in the all CdS/TiO_2_ nanotubes. Compared the main diffraction pattern of three CdS/TiO_2_ nanotubes, the peaks of rutile show an decreased trend due to the addition of Cd^2+^, which mean the rutile may be an intermediate form of CdS/TiO_2_ heterogeneous structure.

### 3.2 Morphology of electrospun nanotubes TiO_2_/CdS

**Fig. 3:**
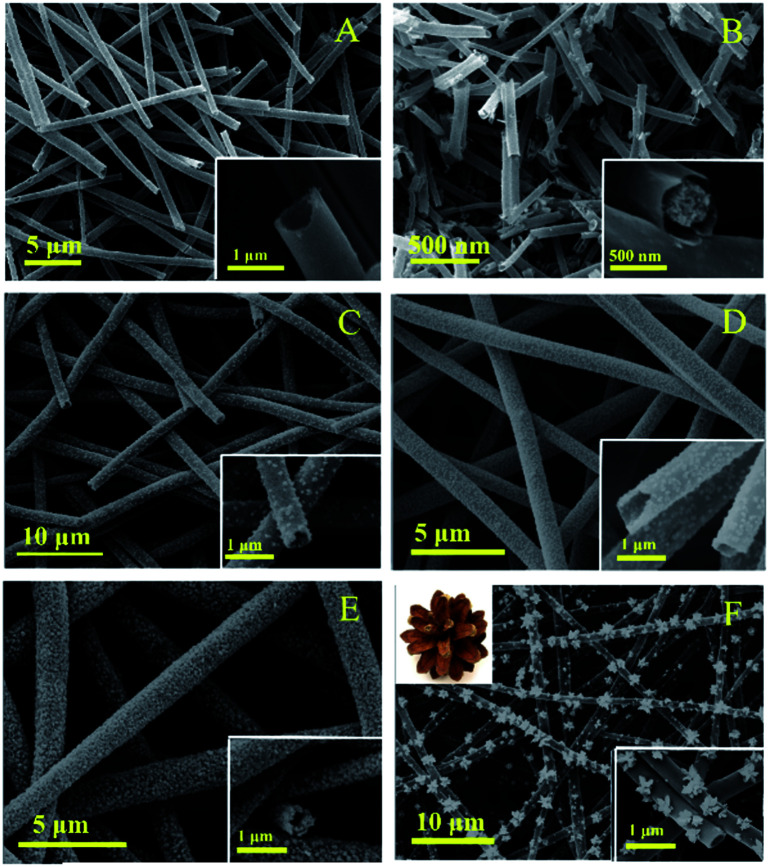
The SEM images of the samples: (A) TiO_2_ nanotubes, (B) core–shell structure TiO_2_ nanotubes, (C) TC1 nanotubes, (D) TC2 nanotubes, (E) TC3 nanotubes and (F) TC4 nanotubes.

**Fig. 4:**
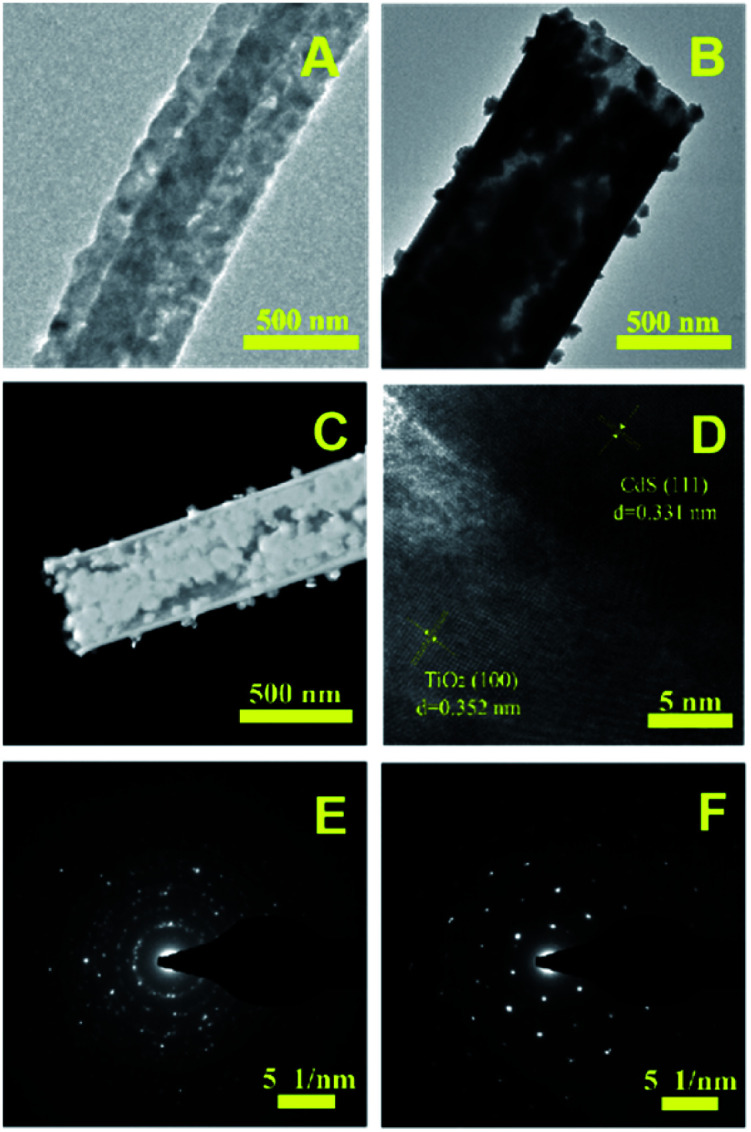
The TEM image of TiO_2_ nanotubes (A) and TC3 nanotubes (B), the STEM image of TC3 nanotubes (C) and the HRTEM of TC3 nanotubes (D), the SAED pattern of the TC3 (E) and the CdS Nps of the surface of TC3 (F).

The morphology of the nanotubes were characterized by SEM. Fig. 3A show the SEM image of TiO_2_, which are composed by regular and uniform nanotubes with diameter of approximately 700 nm and the uniformity was above 80%, demonstrating the TBOT/ethanol solution permeate into the surface of the PS template fibers successful. From the illustration of Fig. 3A, we can observe the high magnification SEM of a TiO_2_ nanotube that present the wall thickness of the nanotube about 100 nm and the inwall of the nanotubes are rough. Fig. 3B clearly present the SEM image of core–shell structure TiO_2_ nanotubes, which was obtained by replacing PS/DMF solution with PAN/DMF solution (PAN: 13 wt%) and other operations were the same. The core might be carbon fibers which produced by the PAN incomplete calcination. The SEM of four kinds of CdS/TiO_2_ hybrid showed in the Fig. 3C–F with low and high magnifications. Compared to the pure TiO_2_ nanotubes, the CdS/TiO_2_ nanotubes could observed amount of nanoparticles deposited on the surface and inwall, and the density of nanoparticles increase with the concentration of Cd^2+^ ions. These results show that the morphology of CdS modified on the nanotubes was related to the solution concentration. Notably, further increase of Cd^2+^, the CdS nanoparticles grown together to form a pine-cone shaped 3D structure as shown in Fig. 3F. The morphology and microstructure of as-prepared photocatalysts also investigated by TEM. The low magnification TEM image (Fig. 4B) further verifies that CdS nanoparticles were well covered the surface and inwall of TiO_2_ nanotubes, compared to the TEM of TiO_2_ nanotubes (Fig. 4A). These nanoparticles with sizes of 5–50 nm are distributed randomly on the TiO_2_. The high resolution TEM image (Fig. 4D) clearly reveals an intimate interface between the TiO_2_ and CdS in the composite to form heterojunctions, which is favorable for the electron transfer in photocatalytic activity. Also demonstrates the CdS/TiO_2_ photocatalyst obtained by hydrothermal method form a heterostructure rather than a mixture of TiO_2_ and CdS. Meanwhile, the high resolution TEM are carried out to survey the lattice fringes of CdS/TiO_2_ nanotubes, the lattice spacing are 0.35 nm and 0.33 nm, which are coincident with the (101) spacing of anatase TiO_2_ and (111) of CdS phase. The legible fringe also suggests the high degree of crystallinity corresponds with the result of XRD results. The Fig. 4E illustrates the selected area electron diffraction (SAED) pattern of TiO_2_/CdS, which presentation a bright diffraction rings assigned to high crystallization and polycrystalline structure. The Fig. 4F was SAED pattern of pure CdS nanoparticles on the surface of TC3 means the obtained CdS particles were pure single crystals.

### 3.3 XPS analysis

**Fig. 5:**
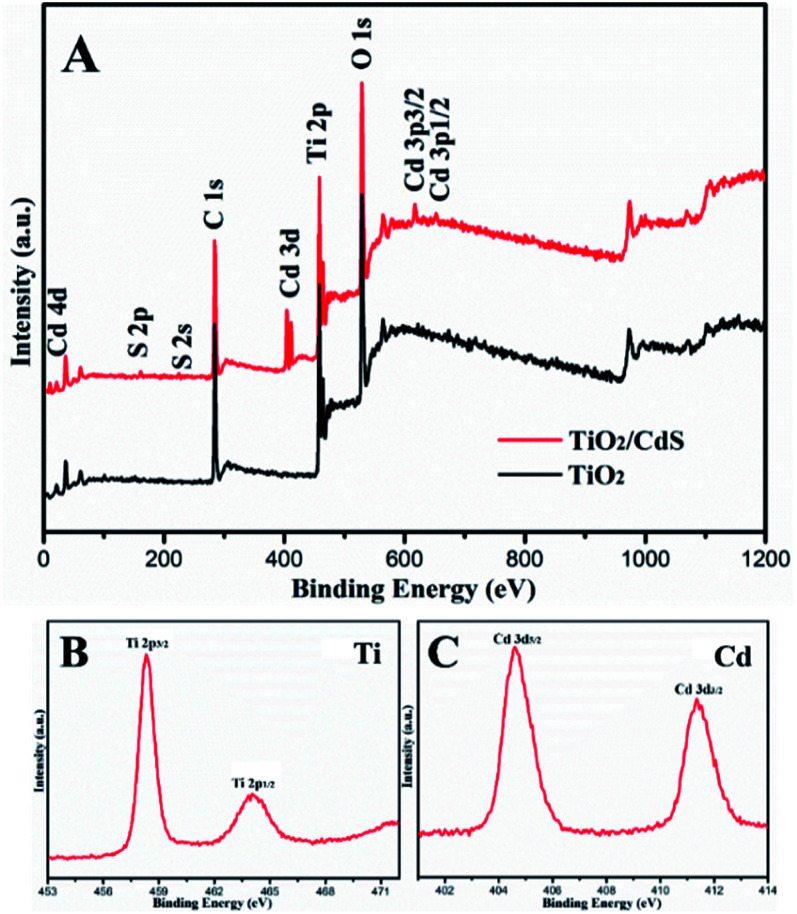
The XPS spectra of the TiO_2_ and TC3 nanotubes: full spectrum of the sample (A), the Ti 2p spectrum (B) and Cd 3d spectrum (C).

XPS analyze could reveal the information about the surface composition and chemical state. As shown in Fig. 5A, CdS/TiO_2_ hybrid (TC2) is mainly composed of five elements, which is Ti, O, Cd, S and C, The peak of C 1s can be ascribed to the hydrocarbon from the instrument itself. The high resolution spectra of Ti 2p were exhibited in the Fig. 5B. The Ti 2p spectra shows the peaks at 458.3 eV and 464.1 eV for Ti 2p_3/2_ and Ti 2p_1/2_ of TiO_2_, respectively. The distance between the two peaks is 5.8 eV, which is identical to that of the neat TiO_2_ indicating that Ti ion is in the form of Ti^4+^, high resolution spectrum of Cd 3d (Fig. 4C) displayed characteristic peaks of 404.5 eV and 411.4 eV, which are corresponding to the Cd 3d_5/2_ and Cd 3d_3/2_ states of Cd^2+^ in CdS, respectively. Consequently, XPS analysis further determines the formation of TiO_2_ and CdS in the sample, which is agreement with XRD and TEM result.

### 3.4 UV-vis diffuse reflection spectra and PL spectra

**Fig. 6:**
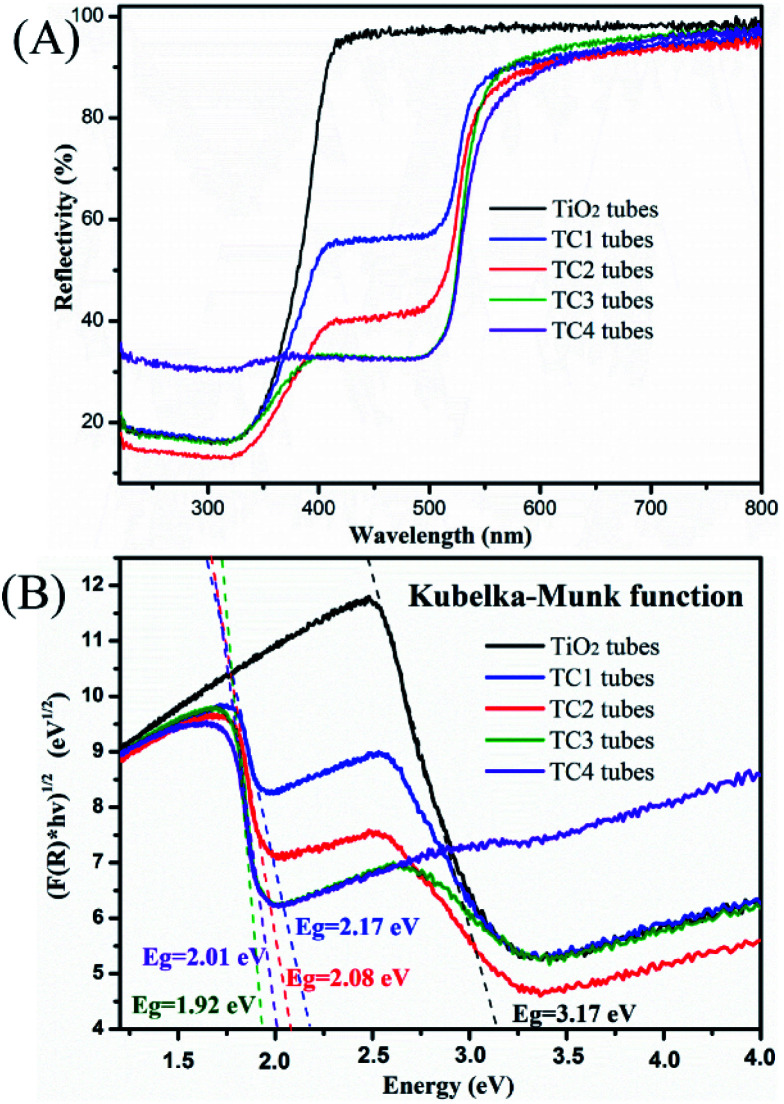
(A) The UV-vis diffuse reflection spectra (DRS) of the as-prepared photocatalysts and (B) Kubelka–Munk transformed reflectance spectra.

UV-vis DRS are tested to determine the optical properties of the photocatalysts. From the results of DRS (Fig. 6A) the reflection spectra of TiO_2_ and CdS/TiO_2_ heterostructure in the region spanning from 230 to 800 nm were showed. Compared to the TiO_2_ nanotubes, the TC2 had a lower reflectivity in the range of 240–380 nm, especially in the ultraviolet light range. After the modified of CdS, all CdS/TiO_2_ nanotubes present a remarkable light adsorption into the visible light region, which may due to the excellent visible-light photoresponse of CdS nanoparticles. Notably, the adsorption edges of CdS/TiO_2_ nanotubes gradually broaden with the mount of modified CdS nanoparticles increasing, indicated the high responsiveness of the photocatalyst to visible light.

Fig. 6B is the forbidden bandwidth obtained by the catalyst according to the Kubelka–Munk formula. Among them, the forbidden band widths of TiO_2_ and four CdS/TiO_2_ heterostructures (TC1, TC2, TC3 and TC4) are 3.17 eV, 2.17 eV, 2.08 eV, 1.92 eV and 2.01 eV. It can be seen from the comparison with the TiO_2_ tubes that the forbidden bandwidth of the CdS/TiO_2_ heterostructure is significantly reduced. Among them, TC3 has the lowest forbidden bandwidth. We can speculate that this sample has the best catalytic performance, and the catalytic experiments proved that the prediction was correct. (The detailed content of the edge position is Fig. S1.†)

### 3.5 Photoluminescence analysis

**Fig. 7:**
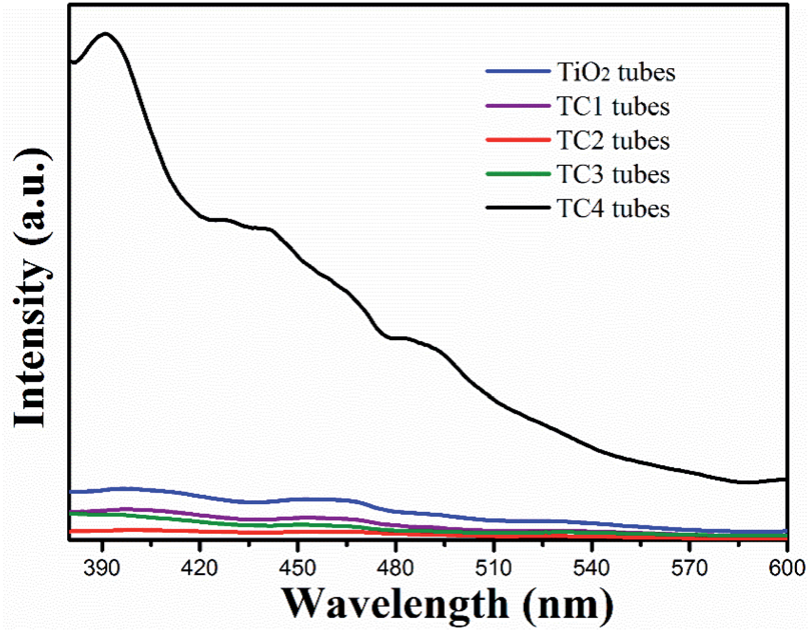
The photoluminescence spectra of the as-prepared photocatalysts.

PL spectroscopy is correlation with the separation and recombination of PG electrons/holes. As shown in Fig. 7, the PL spectra of the TiO_2_ nanotubes showed a strong emission peak at 390 nm (excitation wavelength: 370 nm). However, the PL peak intensity of CdS/TiO_2_ nanotubes was significantly decreased with modification amount of CdS nanoparticles increased. The key factor in the formation of this phenomenon is the defects in the crystal structure of TiO_2_, which act as traps for capturing the photo-excited electrons and thus, inhibit the recombination of the electrons and holes pairs.

### 3.6 Photocatalytic activity and recycled photocatalytic degradation performance

**Fig. 8:**
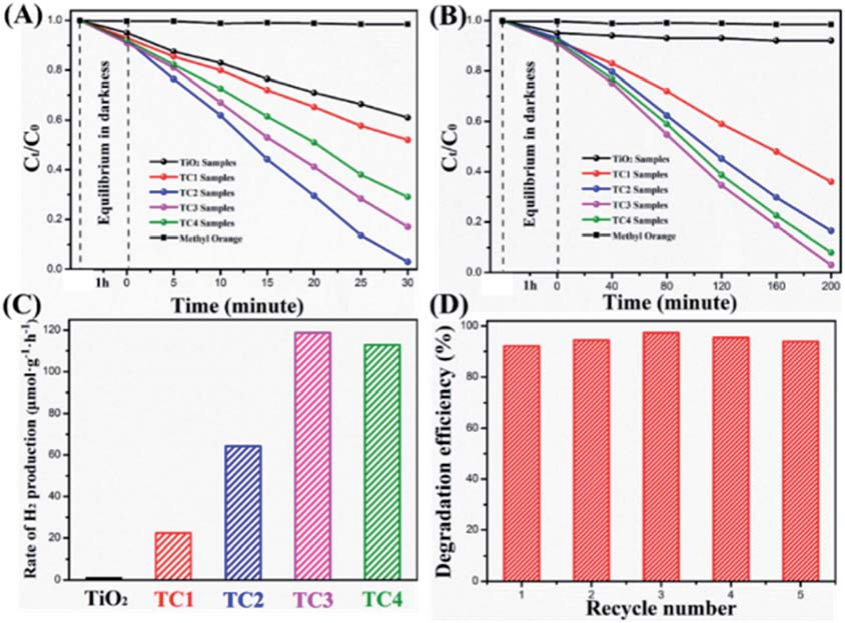
Degradation rates of as-prepared photocatalysts under the UV-light (A); degradation rates of as-prepared photocatalysts under the visible light (B); the rate of H_2_ evolution on TiO_2_/CdS nanotubes under visible light (C); the repeatability tests studied on the TC3 nanotubes for five recycles (D).

To identify the photocatalytic of the as-prepared samples, MO solution degradation were tested under UV and visible irradiation. The photocatalytic effect was evaluated by *C*_0_/*C*_*t*_ (*C*_0_ nd *C*_*t*_ were the initial concentration and the concentration of MO at time *t*, respectively). From the Fig. 8A, shows the concentration change of MO in the process of photodegradation, after irradiated by UV light for 30 min, the photocatalytic degradation efficiencies of the TC2 nanotubes are 95.3% slightly better than the TC3 (83.2%), which is much higher than the TC4 (72.5%) and pure TiO_2_ nanotubes (37.8%). The photodegradation of MO without catalysis almost negligible. However, as shown in Fig. 8B, under visible light irradiation (λ > 420 nm) for 200 minutes the TC3 shows the excellent visible light catalysis (95.2%) similar to the TC4 (94.7%), which CdS load looks like pine-cone shaped and much higher than TC2 (82.6%), TC1 (61.2%), and pure TiO_2_ nanotubes (0.09%), which was consistent with the results of UV-vis DRS. In addition, we also investigated the photocatalytic stability of TiO_2_/CdS heterostructure (TC3) under visible light irradiation. The results indicated that the hybrids exhibited good stability after being reused for five times (Fig. 8D).

Meanwhile, the photocatalytic activities of the nanotubes were evaluated by H_2_ production experiment under the visible light irradiation in aqueous solution (containing 1.0 M Na_2_SO_3_ and 1.0 M Na_2_S as sacrificial reagents). Fig. 8C shows the H_2_ evolution rate by the TiO_2_ and TiO_2_/CdS nanotubes, and there is no appreciable H_2_ was observed when TiO_2_ nanotubes was tested. In contrast, the TC1 exhibited a slightly enhanced H_2_ evolution rate of 22.35 μmol g^−1^ h^−1^. With the increase of CdS, the H_2_ evolution rate are increased. The H_2_ evolution rate of TC2 photocatalyst is 64.26 μmol g^−1^ h^−1^ and TC3 (118.73 μmol g^−1^ h^−1^) reaches a maximum H_2_ evolution rate, demonstrates that is effective to employ CdS as cocatalyst for improving efficiency of TiO_2_ photocatalytic activity. Further increases the amount of modification of CdS, the H_2_ generation rate (TC4 112.86 mol g^−1^ h^−1^) showed a slight decrease. This might be due to the less exposed active sites of TiO_2_/CdS resulting from the excess CdS shielding.

### 3.7 Mechanism for the enhancement of photocatalytic activity

**Fig. 9:**
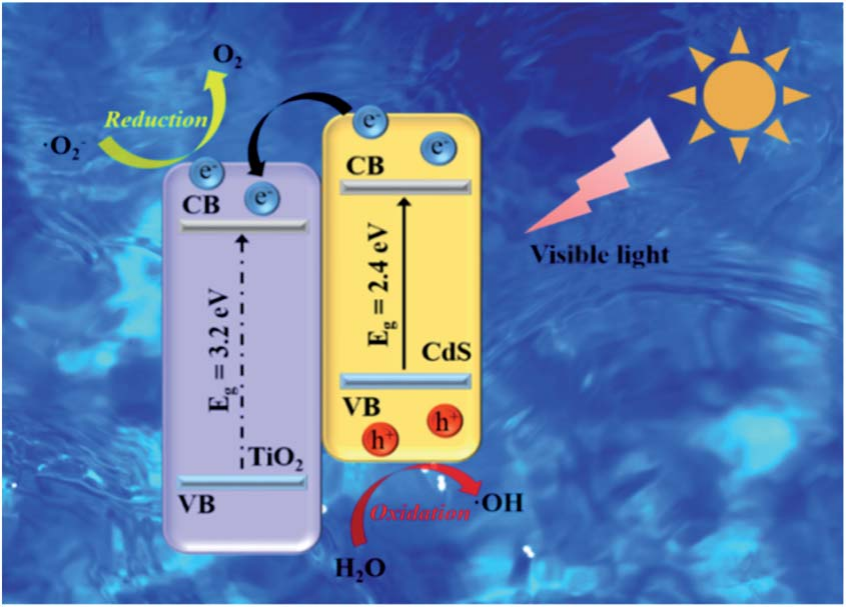
Schematic diagram for the photocatalytic mechanism of the TiO_2_/CdS catalysts under visible-light irradiation.

The principle of photocatalytic decomposition over CdS/TiO_2_ heterostructure is delineated in Fig. 9. Under the visible-light irradiation, the charge carriers were firstly generated from CdS owing to its narrow energy band gap. The photoexcited electrons on the valence band (VB) of CdS can move to their own conduction band (CB). However, the high recombination of electrons (e^−^) and holes (h^+^) of CdS limits its photocatalytic activity. When the TiO_2_ and CdS form a heterostructure, the PG e^−^ on CdS nanoparticles can easily transfer to the CB of TiO_2_ through the interface as a result of the lower conduction edge potential of TiO_2_ than that of CdS. Meanwhile, the h^+^ remain on the VB of CdS. Therefore, the TiO_2_ acts as a temporary e^−^ trap to decrease the recombination of PG e^−^/h^+^. Consequently, the PG h^+^ on the VB of CdS can react with the surface adsorbed H_2_O to generate ·OH. The e^−^ on the CB of TiO_2_ could reduce the adsorbed O_2_ to form ·O_2_^−^. Organic dye (MO) are eventually oxidized by these highly active species to CO_2_ and H_2_O products. The major reaction steps are revealed as follows:

1 TiO_2_/CdS + *hv* → TiO_2_/CdS (e^−^ + h^+^)

2 TiO_2_/CdS (e^−^ + h^+^) → TiO_2_ (e^−^) + CdS (h^+^)

3 e^−^ + O_2_ + → ·O_2_^−^

4 e^−^ + ·O_2_^−^ + H^+^ → ·OH + OH^−^

5 h^+^ + H_2_O → ·OH

6 ·OH + organic → other products → CO_2_ + H_2_O

## 4 Conclusions

In summary, hollow TiO_2_ nanotubes were obtained using electrospinning combined with an impregnation calcination method. CdS nanoparticles were modified on the surface and inner wall of TiO_2_ nanotubes *via* hydrothermal process. By changing the reaction conditions, the size and density of CdS on the surface and inner wall of the TiO_2_ nanotubes can be controlled. Owing to the formation of heterojunction structure, the hybrid nanoparticles could enhanced separation of e^−^/h^+^ pairs and reduce the recombination of charge carriers, and the hollow nanotubes structure provides a larger specific surface area and more active sites, that the CdS/TiO_2_ nanotubes showed remarkably photocatalytic activity than pure TiO_2_. Moreover, that may provide a new method for preparation of heterostructure nanotubes.

## Conflicts of interest

No conflicts of interest in this article.

## Supplementary Material

RA-010-C9RA10895E-s001
