# Supplementary material for: Controllable growth of three-dimensional CdS nanoparticles on TiO2 nanotubes to enhance photocatalytic activity
Source: RSC Adv. 2020 Apr 29;10(28):16776–82. doi: 10.1039/c9ra10895e (PMC9122576; doi:10.1039/c9ra10895e)
Supplement: RA-010-C9RA10895E-s001 [file RA-010-C9RA10895E-s001.pdf]

Electronic Supplementary Material (ESI) for RSC Advances.
This journal is © The Royal Society of Chemistry 2020

Controllable growth of three-dimensional CdS nanoparticles on TiO_2 nanotubes to enhance photocatalytic activity

Guo-Min Liu, [a] Wen-Yuan Jia,[a] Qiu-Shi Jiang,[b] Zhi-Qiang Cheng,*[b]
[a]Department of Orthopedics, the Second Hospital of Jilin University, Changchun 130000, China;
[b]College of Resources and Environment, Jilin Agriculture University, Changchun 130118, China.
*Corresponding Author

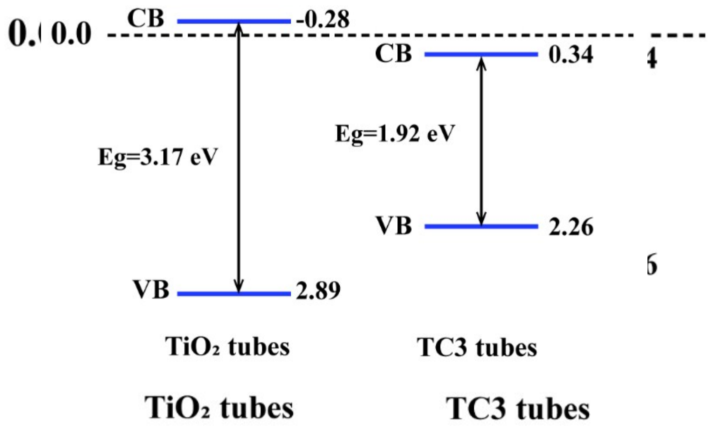

Fig.S1 Calculated band edge (VB, CB) position of TiO_2 tubes and TC3 tubes.

Fig. S1 shows the band edge of the samples TiO_2 tubes and TC3 tubes. The empirical formula for the edge calculation of semiconductors is:

$$E_{CB} = X - E_e - 0.5Eg$$

$$E_{VB} = E_{CB} + Eg$$

E_{CB} is the CB edge potential, and X is the electronegativity of the semiconductor, which is the arithmetic mean of the electronegativity of constituent atoms and first ionization energy. E_e is the energy of free electrons on the hydrogen scale (approximately 4.5 eV). The Eg values of TiO_2 tubes and TC3 tubes are 3.17 eV and 1.92 eV. The band edge position (VB, CB) of TiO_2 and TC3 were 2.89 eV and -0.28 eV, 2.26 eV and 0.34 eV.
